# Anti-Fatigue Activity of Aqueous Extracts of *Sonchus arvensis* L. in Exercise Trained Mice

**DOI:** 10.3390/molecules24061168

**Published:** 2019-03-25

**Authors:** Tian Yuan, Di Wu, Keyu Sun, Xintong Tan, Jia Wang, Tong Zhao, Bo Ren, Beita Zhao, Zhigang Liu, Xuebo Liu

**Affiliations:** Laboratory of Functional Chemistry and Nutrition of Food, College of Food Science and Engineering, Northwest A&F University, Yangling 712100, China; yuantian315@126.com (T.Y.); wd_102016@163.com (D.W.); sunkeyu0201@163.com (K.S.); tanxintong142536@163.com (X.T.); 18392650556@163.com (J.W.); zht3524@163.com (T.Z.); renbo1993@nwafu.edu.cn (B.R.); beitazhao@nwafu.edu.cn (B.Z.); zhigangliu@nwafu.edu.cn (Z.L.)

**Keywords:** *Sonchus arvensis* L., anti-fatigue, lactate, glycogen, exercise

## Abstract

*Sonchus arvensis* L. is a nutritious vegetable and herbal medicine that is consumed worldwide. The aim of this study was to evaluate the anti-fatigue effects and underlying effects of aqueous extract of *Sonchus arvensis* L. (SA). Male C57BL/6 mice from four groups designated vehicle, exercise, exercise with low dose (250 mg/kg) or high dose of SA (500 mg/kg), were trained by swimming exercise and orally administrated with SA every other day for 28 days. The anti-fatigue activity was determined by exhaustive swimming test, as well as the muscle structure, levels of blood hemoglobin, and metabolites including lactate and urea nitrogen. SA alleviated mice fatigue behaviors by eliminating metabolites, while improving muscle structure and hemoglobin levels. Moreover, SA enhanced glycogen synthesis of liver but not muscle via increasing GCK and PEPCK gene expressions. Importantly, SA improved antioxidant enzymes expression and activities in both liver and muscle, which was possibly related to its primary components polysaccharides and the antioxidant components including chlorogenic acid, luteolin, and chicoric acid. Taken together, the anti-fatigue effects of SA could be partly explained by its antioxidant activity and mediating effects on glycogen synthesis and metabolites elimination. Therefore, SA could be a potential nutraceutical for improving exercise performance and alleviating physical fatigue.

## 1. Introduction

Physical fatigue is a complex physiological process, and can be defined as time-dependent exercise and stress-induced difficulty in sustaining regular voluntary activities [1]. There are several theories to explain the underlying mechanisms of physical fatigue after exercise. Firstly, the over-production and accumulation of metabolites such as lactate and urea nitrogen lead to exhaustion of the muscle [2]. Secondly, the oxidative stress and damages induced by the production of reactive oxygen species (ROS) in the muscle or other related organs such as liver [3]. Last but not least, the blood-oxygen concentration balance and the glycogen homeostasis in liver and muscle support sustainability and recovery during and after exercise [4].

The beneficial effects of herbal medicine and nutraceuticals on physical fatigue have been widely investigated in recent decades [1,5,6,7]. These natural products have been found to postpone fatigue and improve exercise performance by eliminating aforementioned metabolites, reducing oxidative stress, or improving glycogen metabolism. *Sonchus arvensis* L. is a traditional food that is common throughout the north of China. It has been investigated that the extract of *Sonchus arvensis* L. possesses bioactivities including antioxidant, hepatoprotective, kidney-protective, and antibacterial effects [8,9,10]. The functional components of *Sonchus arvensis* L. may vary due to different species and extraction methods, however the major fractions are polysaccharide, polyphenol, and flavonoids [11]. Nevertheless, the anti-fatigue effects of *Sonchus arvensis* L. extract and the underlying mechanism are not clear at this stage.

The aim of the present study was to evaluate supplementation of extract of aqueous extract of *Sonchus arvensis* L. (SA) on physical fatigue induced by exhaustive swimming in exercise trained C57BL/6 mice. After the exhaustive swimming test, MRI images and histological tests were performed to examine the structure changes of muscle. The underlying mechanism of SA on physical fatigue were determine the following: the blood routine examination was performed to evaluate the blood-oxygen level and other parameters; the fatigue-related metabolites and redox status parameters were determined in plasma, muscle, and liver; and the glycogen levels and related genes expression were also detected in both muscle and liver.

## 2. Results and Discussion

### 2.1. Effect of SA on Exhaustive Swimming Test in Exercise Trained Mice

**Figure 1:**
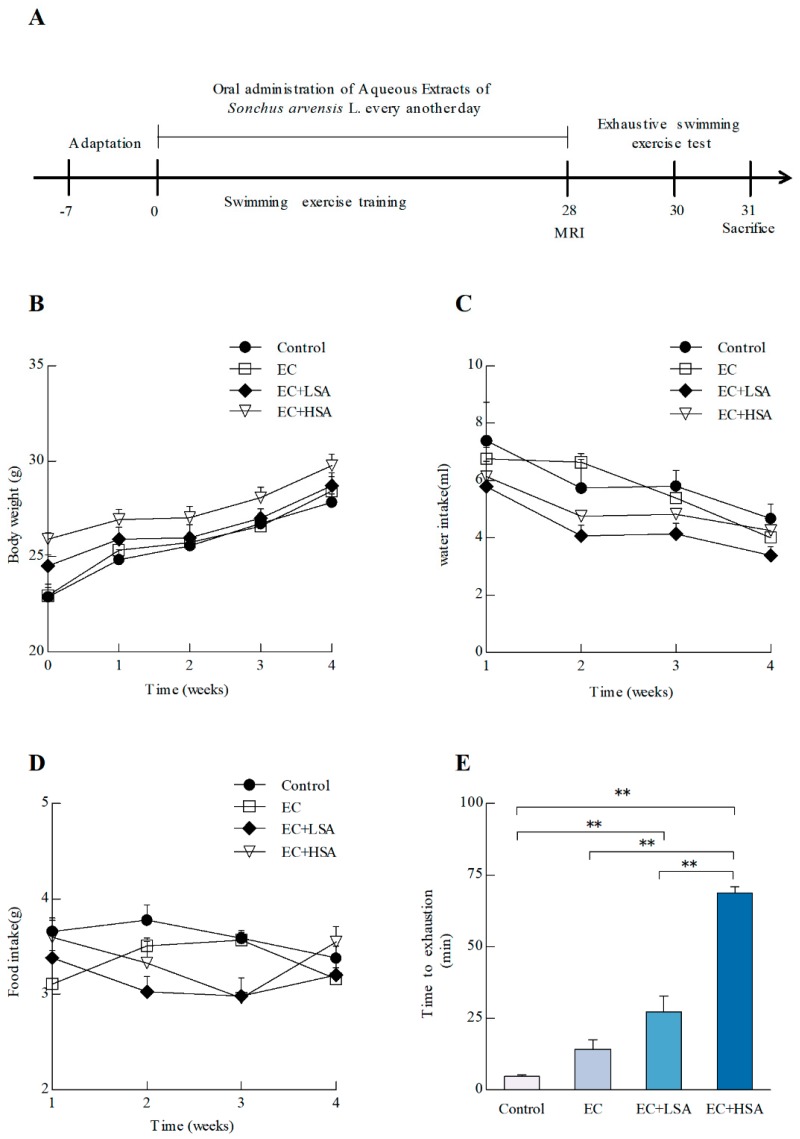
Effects of SA on improving anti-fatigue activity in exercise trained mice. (**A**) Scheme of animal experiments. 8-week old C57BL/6J mice were assigned to 4 groups (*n* = 10) for swimming exercise training or SA supplementation: sedentary control with vehicle treatment (Control); swimming training exercise with vehicle treatment (EC); swimming training exercise with 250 mg/kg SA (EC + LSA) or 500 mg/kg SA (EC + HSA). An exhaustive swimming exercise test was performed for assessments of anti-fatigue activity; (**B**) Body weight trend; (**C**) Water intake trend; (**D**) Food intake trend; (**E**) Time to exhaustion. Data presented as mean ± SEM, *n* = 10, ** *p* < 0.01.

The animal experiments were conducted as Figure 1A. The animals were divided into four groups: vehicle control (control), exercise control (EC), exercise with low dose SA treatment (EC + LSA), and exercise with high dose SA treatment (EC + HSA). The body weights, food intake, and water intake of the mice were measured during the 28-day treatment. As shown in Figure 1B–D, swimming training exercise and SA treatment had no significant influence on body weight, food intake, and water intake changes among four groups (*p* > 0.05). Preliminary experiments also indicated that these doses of SA had no-toxicity during the treatment. After a 28-day swimming training exercise, the exhaustive swimming test was performed. As shown in Figure 1E, the swimming time of EC + LSA and EC + HSA was dramatically longer than the control group and EC group. These results indicated that SA had an effective anti-fatigue activity on the exhaustive swimming test.

We detected the total polyphenols and flavonoids in SA were 82.17 ± 2.15 mg/g and 139.18 ± 7.06 mg/g, respectively. The major components of SA were chlorogenic acid (5.35 ± 3.24 mg/g), luteolin (24.92 ± 5.85 mg/g), chicoric acid (19.84 ± 1.77 mg/g). Chicoric acid, a polyphenol component found in chicory, was demonstrated to possess anti-oxidant and anti-inflammatory effects in our previous research [12,13]. Previous research has found that *Akebia quinata* Decaisne extract, of which chlorogenic acid was the primary component and the mechanism, ameliorated the effects of stress and fatigue-associated brain damage through mechanisms involving regulation of BDNF-TrkB signaling [14]. Lutein has also been reported to improve exercise performance and skeletal muscle contractile function during ischemia/reperfusion [15].

The total polysaccharide was 64.8 ± 0.74 mg/g. The anti-fatigue potential of some polysaccharides from plants has also been well-reported [16,17,18]. However, the structure of polysaccharides is very complicated, and thus its in vivo bioavailability and bioactivities are unclear at present. The potential anti-fatigue effects might be also related to its effects on gut microbiota and gut barrier function [19]. Therefore, the structure of polysaccharides and the underlying structure-activity mechanism should be clarified in a future study. Besides, the long-term treatment of SA on exercise trained mice is also needed to investigate if there are any side-effects of SA treatment.

### 2.2. Histological Examination on Mice Hind Leg Muscle Structure in Exercise Trained Mice

**Figure 2:**
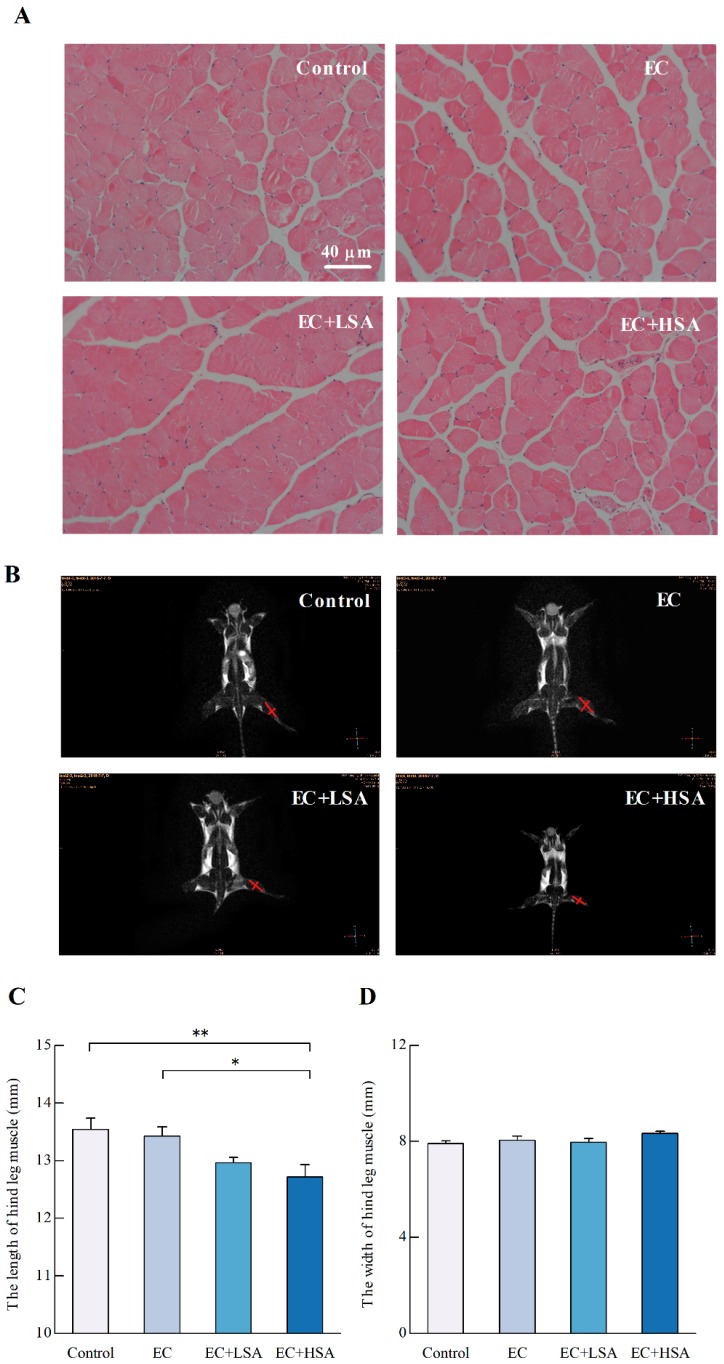
Histological examination on mice hind leg muscle structure in exercise trained mice. (**A**) H&E staining results; (**B**) MRI results; (**C**) hind limb thigh muscle length (mm); (**D**) hind leg skeletal muscle; All data are expressed as the mean ± SEM (*n* ≥ 3). * *p* < 0.05, ** *p* < 0.01.

The histological changes of hind leg skeletal muscle were observed by H&E staining and Magnetic resonance imaging (MRI). As shown in Figure 2A, there were no significant differences in histological observations of any of the target tissues among all four groups.

MRI is a medical imaging technology, which is used to image the anatomical and physiological processes of the body and healthy diseases [20]. The length and width of gastrocnemius muscle in mice hind limbs were measured by MRI imaging. As shown in Figure 2B–D, the effect of SA on the width of hind limb muscle in mice have no significance, however, the hind limb muscle length was significantly affected by high dose of intake (*p* < 0.05). The results demonstrated that the high dose intake of SA could change the length of hind limb muscle, but had no significant effect on the width, which indicated that the anti-fatigue effects of SA could be possibly related to the muscle structure changes.

### 2.3. Effects of SA on Blood-Oxygen Related Parameters in Exercise Trained Mice

As shown in Table 1, the blood routine of four groups mice were performed. The results demonstrated that a high-dose of SA significantly increased hemoglobin levels in treated mice compared to the EC group. Hemoglobin is responsible to carry oxygen through blood to all parts of the body. Therefore, balanced hemoglobin levels are essential for maintaining different organs energy metabolism [21]. These results indicated that SA might have beneficial effects on alleviating fatigue by improving blood-oxygen.

### 2.4. Effects of SA on Liver and Muscle Glycogen Levels

**Figure 3:**
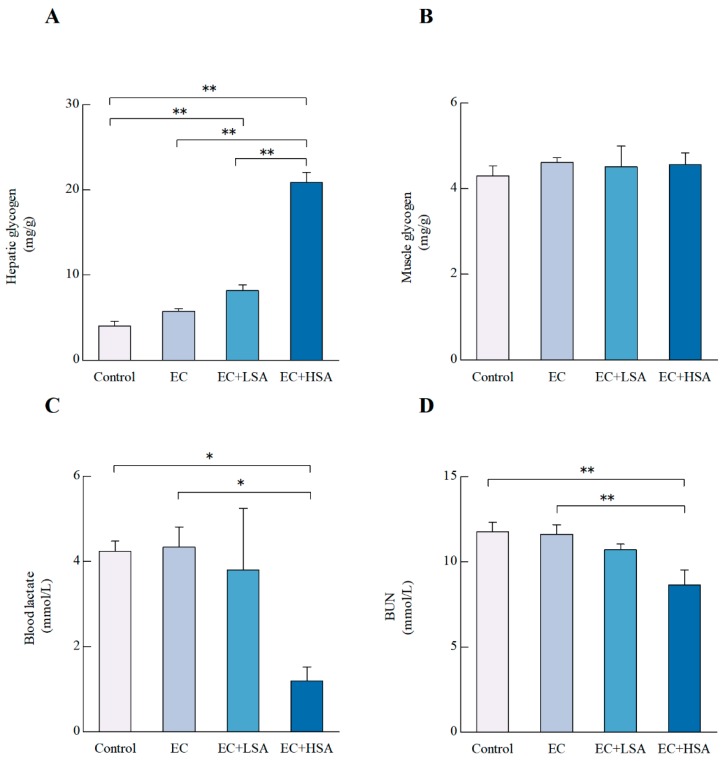
Effects of SA on hepatic glycogen, muscle glycogen, blood lactate and BUN levels in exercise trained mice. (**A**) Hepatic glycogen; (**B**) Muscle glycogen; (**C**) Blood lactate; (**D**) BUN level. Data presented as mean ± SEM, *n* = 6. * *p* < 0.05, ** p < 0.01.

Blood glucose uptake and muscle glycogen breakdown provide the initial energy for exercise, and then energy is supplied by the liver glycogen breakdown [22]. Therefore, the level of glycogen in liver and muscle are both significant makers related to fatigue [23]. In the present study, the hepatic glycogen levels were up-regulated by the high dose of SA supplementation compared with EC group (*p* < 0.01) (Figure 3A). However, there were no significant differences of muscle glycogen levels among these groups (Figure 3B).

Glycolysis, the main energy source for fierce exercise in a short time, and blood lactate is the glycolysis product of carbohydrate in the condition of anaerobic. Therefore, there is a close relationship between blood lactate and workload intensity, and blood lactate is one of the important parameters of the exercise intensity or the fatigue degree [23]. In this study, a high dose of SA significantly suppressed the contents of blood lactate (*p* < 0.05) (Figure 3C). Besides, it has also been reported that blood urine nitrogen (BUN) causes fatigue [24]. High dose of SA suppressed the contents of BUN (*p* < 0.01) compared with group EC (Figure 3D).

### 2.5. Effects of SA on the Glycogen Synthesis Related Gene Expressions in Mice Liver and Muscle

**Figure 4:**
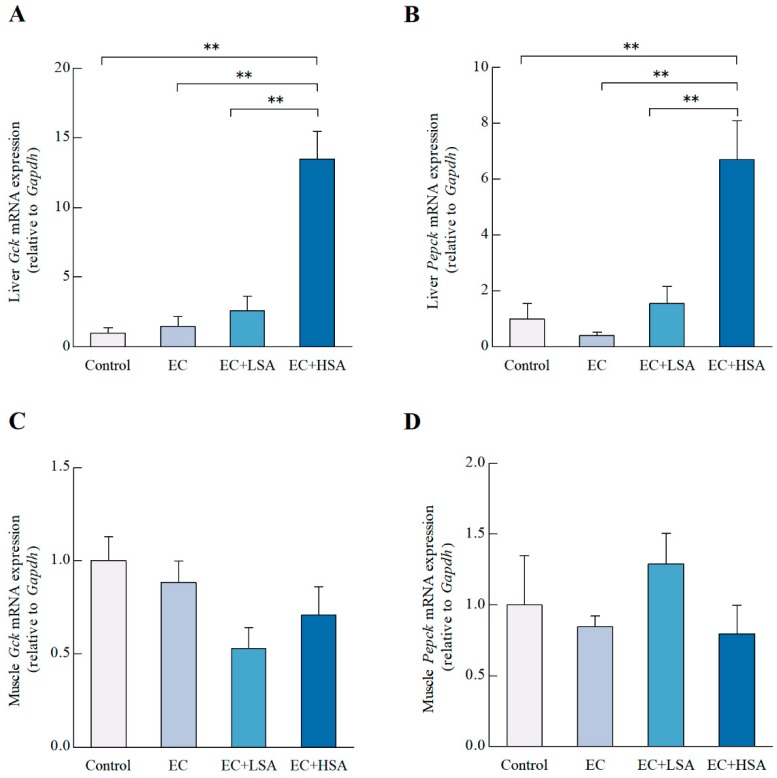
Effects of SA on glycogen synthesis in exercise trained mice. The mRNA expressions of (**A**) *Gck*, (**B**) *Pepck* in mice liver; The mRNA expressions of (**C**) *Gck*, (**D**) *Pepck* in mice muscle. Data presented as mean ± SEM, *n* = 6, ** *p* < 0.01.

The glucokinase (GCK) acts as an eminent player in regulating the glucose metabolism and homeostasis [25]. In the current study, we studied the effects of SA supplementation on the mRNA expression of *Gck* in mice liver and muscle. As illustrated in Figure 4A, high dose of SA significantly increased the mRNA expression of *Gck* (*p* < 0.01) compared with EC group in mice liver. However, there existed no significant difference of *Gck* mRNA expression in mice muscle among these groups (Figure 4C).

Hepatic gluconeogenesis compensates for discontinuities in nutrient supply to maintain systemic energy homeostasis. Phosphoenolpyruvate carboxykinase (PEPCK) is an essential enzyme regulating gluconeogenesis by converting oxaloacetate into CO_2_ and phosphoenolpyruvate [26]. The PEPCK in skeletal muscle have multiple biological functions, including: providing pyruvate for the synthesis of alanine by alanine aminotransferase [27]. In the present study, high dose of SA significantly increased the mRNA expression of *Pepck*PEPCK (*p* < 0.01) in mice liver compared with EC group (Figure 4B). However, in terms of the of *Pepck* mRNA expression in muscle, there existed no significant difference among the four groups (Figure 4D).

### 2.6. SA Ameliorated Oxidative Stress in Mice Liver and Muscle

**Figure 5:**
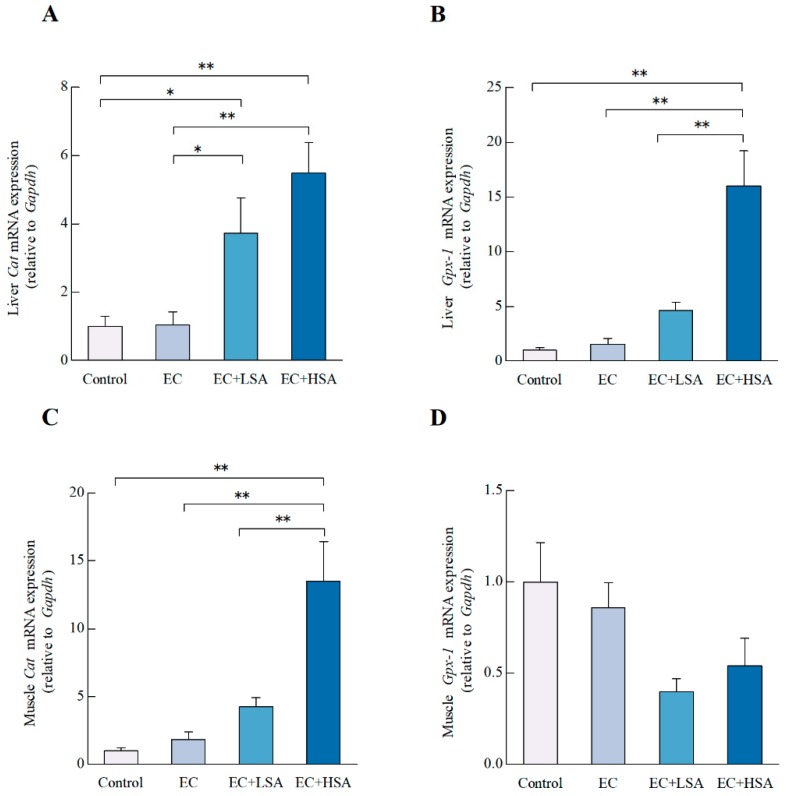
Effects of SA on antioxidant gene expressions in exercise trained mice. The mRNA expressions of (**A**) *Cat*, (**B**) *Gpx-1* in mice liver; The mRNA expressions of (**C**) *Cat*, (**D**) *Gpx-1* in mice muscle. Data presented as mean ± SEM, *n* = 6. * *p* < 0.05, ** *p* < 0.01.

Reactive oxygen species (ROS) plays an essential role during human normal physiological and pathological process such as exercise and metabolism [28]. Over production of ROS unbalanced cellular redox status and led to tissues damage [29]. Importantly, excessive oxidative stress can be elicited by strenuous exercise and further lead to irreversible oxidize damage [30]. The cellular major defending antioxidant enzymes including glutathione peroxidase (GPx), superoxide dismutase (SOD), and catalase (CAT) can prevent biomacromolecules such as DNA, protein, and lipids from attacks of ROS [31]. Malondialdehyde (MDA) is an end-product of lipid peroxidation, which is also a vital biomarker of oxidative stress [32]. High dose of SA enhanced the activity of SOD (*p* < 0.05) in mice liver compared with EC group (Table 2). Meanwhile, both low dose (*p* < 0.05) and high dose of SA (*p* < 0.01) markedly elevated the mRNA expression of *Cat* in mice liver (Figure 5A) and also increased the *Gpx-1* mRNA expression (*p* < 0.01) (Figure 5B) in mice liver compared with EC group. High dose of SA down-regulated the activity of MDA (*p* < 0.05) (Table 3) and increased the mRNA expression of *Cat* (*p* < 0.01) in mice muscle compared with EC group (Figure 5C). In terms of the *Gpx-1* mRNA expression in muscle, there were no significant differences between these groups (Figure 5D). The main components of the extract include chlorogenic acid, chicoric acid and Luteolin (Table 3). Our previous study indicated that chicoric acid could activate Nrf2 signaling and increased the expressions of HO-1 and NQO-1 in both microglial cells and the brain of LPS-treated mouse [12]. Chlorogenic acid was reported has the ability to mitigate ROS and related negative effects related to oxidative stress [33]. Luteolin against oxidative stress through increasing the HO-1 and glutamate cysteine ligase in rat primary hepatocytes [34]. However, considering the muscle glycogen did not altered in SA treatment groups and we only detected serum lactate and BUN levels in this study, the lactate accumulation in muscle should be observed in future research to fully understand the mechanisms of SA on muscle fatigue.

## 3. Methods and Materials

### 3.1. Preparation of SA and Determination of Major Components

The procedure of the aqueous extract of *Sonchus arvensis* L. were performed as following description. Briefly, fresh *Sonchus arvensis* L. leaves were clean up and then incubated 50 °C for 24 h until completely dehydrated. The dried *Sonchus arvensis* L. leaves were crushed with a superfine grinder and sifted through 40 meshes. The *Sonchus arvensis* L. dry powder were diluted in the water in a radio of 1:20, and then extracted under 100 °C for 60 min. After filtration, the extract was concentrated to 30 mL by rotary evaporator and then freeze-dried for 36 h by using a vacuum freeze-dryer (Sihuan Scientific Instrument Factory Co., Ltd., Beijing, China).

The total polysaccharide of SA was measured by the phenol-sulphuric acid method as described in previous research. Initially, A 2 mL of diluted extract of SA and 1mL of 5% phenol were mixed in a tube. Then, 5 mL of concentrated H_2_SO_4_ was added and left to react for 5 min. After that, the mixture was kept for 30 min at 55 °C. The absorbance of the mixture was measured at 490 nm with a UV-vis spectrophotometer (UVmini-1240, Shimadzu, Kyoto, Japan). The content of total polysaccharide was calculated by standard curve of glucose and results are expressed as equivalents of glucose (mg/g) [35].

The analysis of the total polyphenols and flavonoids in the *Sonchus arvensis* L. were performed as previously described [36,37]. The concentration of chlorogenic acid, luteolin, and chicoric acid were measured by HPLC (Shimadzu, Kyoto, Japan) with chromatographic column C18 (5 μm, 4.6 × 250 mm, Agilent, Waldbronn, Germany). The detector was SPD-15C UV/VIS detector. The detection wavelength was 220 nm. The mobile phase consisted of 0.05% formic acid (A) and 83% acetonitrile (B) was performed as follow: 0–5 min (15% B); 5–23 min (70% B); 23–23.10 min (10% B). The chromatographic fingerprints of these compounds were as shown in Supplemental Materials.

As shown in Table 3, the total polyphenols and flavonoids were 82.17 ± 2.15 mg/g and 139.18 ± 7.06 mg/g, respectively. The major components of SA were chlorogenic acid (5.35 ± 3.24 mg/g), luteolin (24.92 ± 5.85 mg/g), chicoric acid (19.84 ± 1.77 mg/g).

### 3.2. Animal Experimental Design

8-week old male C57BL/6J mice (purchased from Beijing Vital River Laboratory Animal Technology Co., Ltd., Beijing, China) were housed in the Northwest A&F University animal facility under standard conditions (12/12 light-dark cycle, temperature 22 ± 2 °C, humidity at 50 ± 15%). Animals were randomly divided to 4 different groups (*n* = 10): sedentary control with vehicle treatment (Control); swimming training exercise with vehicle treatment (EC); swimming training exercise with low dose (250 mg/kg bodyweight, EC + LSA) or high dose (500 mg/kg bodyweight) of SA (EC + HSA). Mice were orally administrated with SA every another day for 4 weeks. The vehicle group received the same volume of sterile water. Animal experiments were performed and approved by the animal ethics committee of Northwest A&F University.

### 3.3. Swimming Training Exercise and Exhaustion Exercise Test

Animals in the EC, EC + LSA and EC + HSA groups underwent an intensive aerobic swim training for 20 min during the 28-day treatment. The swimming training were performed after gavage treatment, the time interval was 30 min. The swimming exercise was carried out in a circle plastic pool (R = 8 cm) 30 cm deep with water (25 ± 2 °C).

We performed the exhaustive swimming test after 30 min of the last gavage. The mice were given an added-weight equal to 5% body weight to evaluate endurance. The mice endurance was recorded at the beginning time of exhaustion, which was determined by observing the loss of coordinated movements and failure to return to the surface within 8 s. The animals were euthanized and all serum and tissue samples were collected immediately after the exhaustion exercise test.

### 3.4. Hematoxylin and Eosin (H&E) Staining and MRI

Fixed the muscle tissue in 4% (*v*/*v*) paraformaldehyde/PBS and embedded it in paraffin, then stained the tissue with hematoxylin and eosin (H&E), finally observed by light microscopy (Olympus, Tokyo, Japan) (×200). For MRI (Philips, Amsterdam, Netherlands) (1.5T), after 30 min of intragastric administration of SA, each mouse was given a swimming exercise for 20 min, then the mice were dried and anesthetized. The mice were then laid flat on the wooden board, their limbs and trunk were fixed with tape, they were placed in the nuclear magnetic resonance testing room, and we selected the third cross-section for measuring the length and width of the hind leg muscle.

### 3.5. Routine Blood Tests

The mice routine blood tests including white blood cell, lymphocyte, lymphocyte (%), monocyte, monocyte (%), red blood cell, hemoglobin, hematocrit, and platelet were measured by Beckman Coulter AU680 automatic biochemical analyzer (Beckman Coulter, Inc., Brea, CA, USA).

### 3.6. Analyses of Antioxidant Enzyme Activity of Mice Liver and Muscle

The total superoxide dismutase (SOD, A001-3), catalase (CAT, A007-1-1) activity and malondialdehyde (MDA, A003-1) contents were performed according to the enzymatic assay kits instructions. (Nanjing Jiancheng Bioengineering Institute, Nanjing, China).

### 3.7. Quantitative Real-Time Polymerase Chain Reaction Analyses

The total RNAs of liver and muscle tissue were isolated by RNA Extraction Kit (TaKaRaMiniBEST RNA Extraction Kit, Dalian, China) following the manufacturer’s instructions and then reverse-transcribed (RT) into cDNA by reverse transcription kit (TaKaRa PrimeScript™ RT Master Mix, Dalian, China). The mRNA expressions were quantified using PCR kit (TaKaRa SYBR^®^ Premix Ex Taq™II, Dalian, China) and the real-time detection was performed by real time system (CFX96™, Bio-Rad, Hercules, CA, USA). The primers employed in current study listed in Table 4. Results were normalized to GAPDH mRNA expression levels, and the 2^−ΔΔCt^ method was used to calculate the relative gene expression.

### 3.8. Statistical Analysis

Data were presented as means ± SEM. Significant differences between measurements among groups were analyzed using one-way factorial analysis of variance (ANOVA), followed by Tukey’s test (software, Graphpad Prism 6, GraphPad Software Inc., San Diego, CA, USA). Results were considered to be statistically significant if *p* < 0.05.

## 4. Conclusions

To summarize, the present study indicated that aqueous extract of *Sonchus arvensis* L. has anti-fatigue activity by alleviating exhaustive swimming-induced oxidative stress, reducing plasma metabolites including lactate and ammonia, and improving the muscle structure and liver glycogen synthesis and reserve. Therefore, the aqueous extract of *Sonchus arvensis* L. could be a promising nutraceutical supplementation in elevating exercise performance and tolerance, and in reducing physical fatigue in exhaustive exercise and modern life stress. Further studies of the mechanisms of the extract major components on eliminating ROS and metabolites are also necessary to clarify the intracellular target of these components.

## Figures and Tables

**Table 1:** Effects of *Sonchus arvensis* L. extract on routine blood of mice.

Routine Blood Test	Control	EC	EC + LSA	EC + HSA
White blood cell (10^9^/L)	3.72 ± 0.74 ^1^	3.02 ± 1.04	2.72 ± 0.87	2.56 ± 1.30
Lymphocyte (10^9^/L)	2.54 ± 0.54	2.35 ± 0.64	2.03 ± 0.68	2.00 ± 1.01
Lymphocyte (%)	69.54 ± 7.69	79.7 ± 8.42	74.14 ± 4.82	78.26 ± 5.71
Monocyte (10^9^/L)	0.10 ± 0.03	0.07 ± 0.04	0.17 ± 0.06	0.062 ± 0.02
Monocyte (%)	2.62 ± 0.52	2.06 ± 0.92	2.04 ± 1.06	2.84 ± 1.55
Red blood cell (g/L)	7.74 ± 0.21	8.06 ± 0.83	8.54 ± 0.56	8.21 ± 0.48
Hemoglobin (10^9^/L)	112.50 ± 2.65	111.33 ± 8.08	129.67 ± 6.51	124.33 ± 4.16 *
Hematocrit (%)	0.38 ± 0.01	0.40 ± 0.04	0.42 ± 0.03	0.41 ± 0.02
Platelet (10^9^/L)	834.40 ± 154.77	842.50 ± 83.24	807.60 ± 75.48	749.75 ± 77.48

^1^ All data are expressed as the mean ± SEM (*n* ≥ 6). * *p* < 0.05 versus EC group.

**Table 2:** Effects of *Sonchus arvensis* L. extract on antioxidant enzyme activity in mice liver and muscle.

Antioxidant Enzymes	Control	EC	EC + LSA	EC + HSA
**liver**				
SOD (U/g prot) ^1^	193.76 ± 3.67 ^2^	193.66 ± 22.41	203.64 ± 27.92	248.09 ± 13.89 *
CAT (U/mL)	45.29 ± 8.99	61.58 ± 0.80	86.37 ± 13.90	82.28 ± 5.25
MDA (nmol/g prot)	30.53 ± 6.62	26.42 ± 4.99	22.21 ± 3.14	20.09 ± 8.48
**muscle**				
SOD (U/g prot)	94.73 ± 13.43	104.49 ± 12.29	102.11 ± 15.59	97.98 ± 5.83
CAT (U/mL)	2.46 ± 1.64	2.71 ± 0.79	2.63 ± 0.19	2.67 ± 1.98
MDA (nmol/g prot)	36.85 ± 1.48	42.42 ± 9.11	36.17 ± 8.21	25.36 ± 0.69 *

^1^ prot stands for protein. ^2^ All data are expressed as the mean ± SEM (*n* ≥ 6). * *p* < 0.05 versus EC group.

**Table 3:** Major bioactive components content.

Major Components	Contents (mg/g)
Total polysaccharide	64.8 ± 0.74 ^1^
Total polyphenols	82.17 ± 2.15
Total flavonoids	139.18 ± 7.06
Chlorogenic acid	5.35 ± 3.24
Luteolin	24.92 ± 5.85
Chicoric acid	19.84 ± 1.77

^1^ All data are expressed as the mean ± SEM (*n* = 3).

**Table 4:** Primer sequences used for RT-qPCR analysis.

	Forward Primer	Reverse Primer
*Gck*	AGTATGACCGGATGGTGGATGAA	CCAGCTTAAGCAGCACAAGTCGTA
*Pepck*	ACTGTTGGCTGGCTCTCACTG	GGGAACCTGGCGTTGAATGC
*Cat*	CGTTCGATTCTCCACAGTCA	CCCACAAGATCCCAGTTACC
*Gpx-1*	AAGGCTCACCCGCTCTTTAC	ACACCGGAGACCAAATGATG
*Gapdh*	TGGAGAAACCTGCCAAGTATGA	TGGAAGAATGGGAGTTGCTGT

## Supplementary Materials

The following are available online, Figure S1: The representative HPLC chromatographic fingerprints of aqueous extracts of *Sonchus arvensis* L. (**A**) The chromatograms of standard luteolin; (**B**) The chromatograms of standard chicoric acid; (**C**) The chromatograms of standard chlorogenic acid; (**D**) The chromatograms of aqueous extracts of *Sonchus arvensis* L.
