# Supplementary material for: Anti-Fatigue Activity of Aqueous Extracts of Sonchus arvensis L. in Exercise Trained Mice
Source: Molecules. 2019 Mar 25;24(6):1168. doi: 10.3390/molecules24061168 (PMC6470720; doi:10.3390/molecules24061168)
Supplement: Supplementary file 1 [file molecules-24-01168-s001.pdf]

Supplemental Figure 1

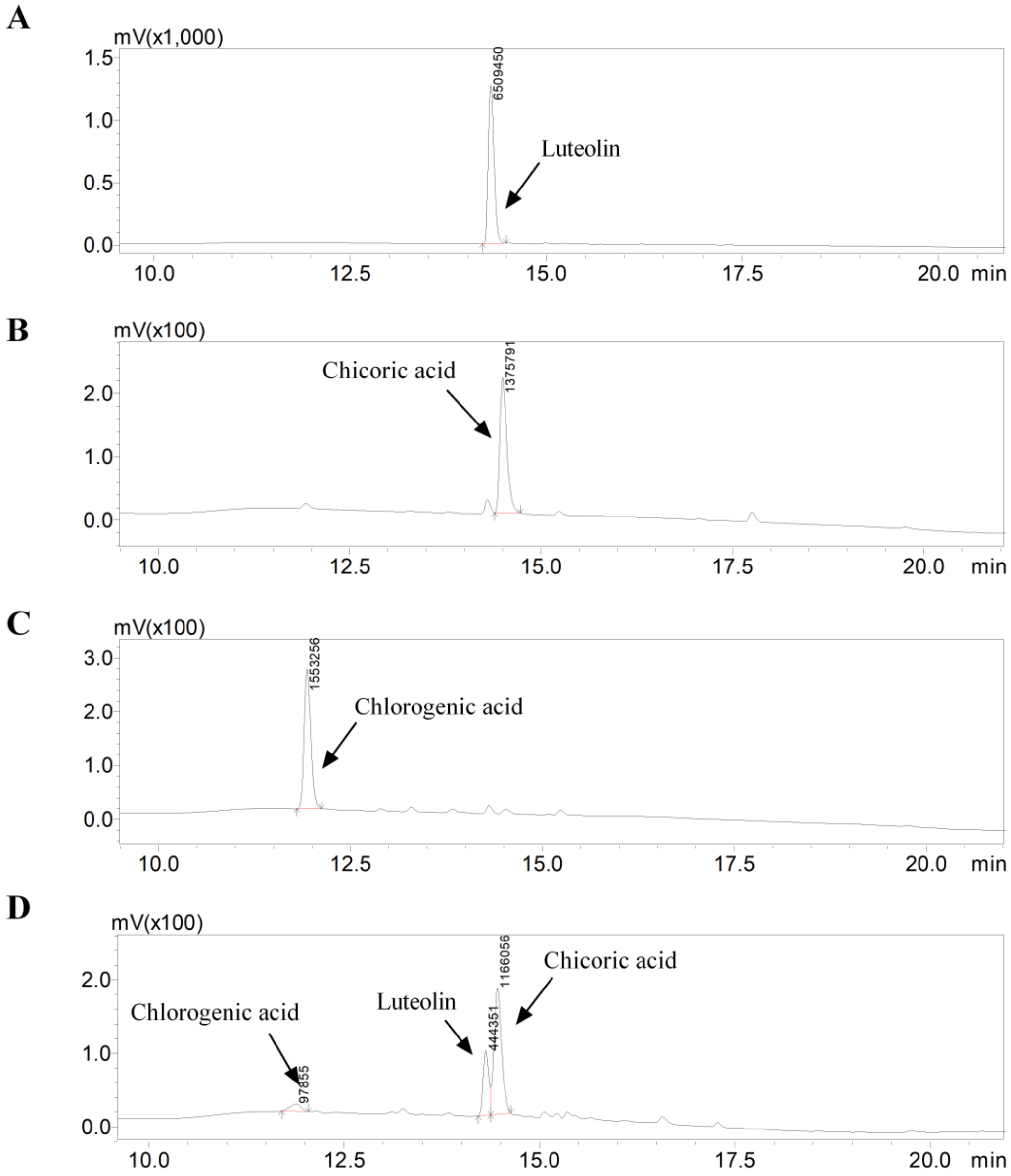

Supplemental Figure 1 The representative HPLC chromatographic fingerprints of aqueous extracts of *Sonchus arvensis* L. (A) The chromatograms of standard luteolin; (B) The chromatograms of standard chicoric acid; (C) The chromatograms of standard chlorogenic acid; (D) The chromatograms of aqueous extracts of *Sonchus arvensis* L.
